# Depressive symptoms and all-cause mortality among middle-aged and older people in China and associations with chronic diseases

**DOI:** 10.3389/fpubh.2024.1381273

**Published:** 2024-05-22

**Authors:** Lan Zhu, Yixi Wang, Jiaqi Li, Huan Zhou, Ningxiu Li, Yuanyuan Wang

**Affiliations:** ^1^School of Education and Psychology, Key Research Institute of Humanities and Social Sciences of State Ethnic Affairs Commission, and Research Centre of Sichuan Minzu Education Development, Southwest Minzu University, Chengdu, China; ^2^West China School of Public Health and West China Fourth Hospital, Sichuan University, Chengdu, Sichuan, China; ^3^Key Laboratory of Brain, Cognition and Education Sciences, Ministry of Education, China; School of Psychology, Center for Studies of Psychological Application, and Guangdong Key Laboratory of Mental Health and Cognitive Science, South China Normal University, Guangzhou, China

**Keywords:** depressive symptoms, all-cause mortality, middle-aged people, older people, chronic diseases

## Abstract

**Introduction:**

It remains unclear whether depressive symptoms are associated with increased all-cause mortality and to what extent depressive symptoms are associated with chronic disease and all-cause mortality. The study aims to explore the relationship between depressive symptoms and all-cause mortality, and how depressive symptoms may, in turn, affect all-cause mortality among Chinese middle-aged and older people through chronic diseases.

**Methods:**

Data were collected from the China Health and Retirement Longitudinal Study (CHARLS). This cohort study involved 13,855 individuals from Wave 1 (2011) to Wave 6 (2020) of the CHARLS, which is a nationally representative survey that collects information from Chinese residents ages 45 and older to explore intrinsic mechanisms between depressive symptoms and all-cause mortality. The Center for Epidemiological Studies Depression Scale (CES-D-10) was validated through the CHARLS. Covariates included socioeconomic variables, living habits, and self-reported history of chronic diseases. Kaplan–Meier curves depicted mortality rates by depressive symptom levels, with Cox proportional hazards regression models estimating the hazard ratios (HRs) of all-cause mortality.

**Results:**

Out of the total 13,855 participants included, the median (*Q*1, *Q*3) age was 58.00 (51.00, 63.00) years. Adjusted for all covariates, middle-aged and older adults with depressive symptoms had a higher all-cause mortality rate (HR = 1.20 [95% CI, 1.09–1.33]). An increased rate was observed for 55–64 years old (HR = 1.23 [95% CI, 1.03–1.47]) and more than 65 years old (HR = 1.32 [95% CI, 1.18–1.49]), agricultural Hukou (HR = 1.44, [95% CI, 1.30–1.59]), and nonagricultural workload (HR = 1.81 [95% CI, 1.61–2.03]). Depressive symptoms increased the risks of all-cause mortality among patients with hypertension (HR = 1.19 [95% CI, 1.00–1.40]), diabetes (HR = 1.41[95% CI, 1.02–1.95]), and arthritis (HR = 1.29 [95% CI, 1.09–1.51]).

**Conclusion:**

Depressive symptoms raise all-cause mortality risk, particularly in those aged 55 and above, rural household registration (agricultural Hukou), nonagricultural workers, and middle-aged and older people with hypertension, diabetes, and arthritis. Our findings through the longitudinal data collected in this study offer valuable insights for interventions targeting depression, such as early detection, integrated chronic disease care management, and healthy lifestyles; and community support for depressive symptoms may help to reduce mortality in middle-aged and older people.

## 1 Introduction

Depression is a serious mental illness that affects mood, thinking, and physical functioning (1, 2). Depressive symptoms are characterized by persistent low mood, loss of interest, sleep disturbances (3–5), persistent and unrelieved fatigue, loss of appetite, and physical discomfort (6–8). The persistence and exacerbation of these depressive symptoms are important causes and diagnostic criteria for determining and progressing depression (9, 10). Depression significantly increases mortality risk and reduces life expectancy, with a mortality rate increment ranging from 15 to 25% (11). A Danish cohort study done in the year from 1995 to 2013 revealed a life expectancy reduction of 14.0 years for men and 10.1 years for women with depression compared to the general population (12). The global prevalence of depression increased by 28% in 2020, with projections indicating that it will be the leading global disease burden by 2030 (13).

The persistence and exacerbation of depressive symptoms is not only an important symptomatic criterion for the diagnosis of depression (14), but these symptoms are important causes of deterioration in physical health (15, 16), impact on immune function (17, 18), increasing the risk of chronic disease (19, 20), and self-injurious suicidal behavior (21, 22). There is a long-term and complex relationship between depressive symptoms, mortality risk, and life expectancy (23, 24). It has been suggested that depressive symptoms may induce a chronic stress response (25), likely due to the interplay of various factors, including traumatic events in life (26), a deficiency in social support (27), and adverse lifestyle habits (28). This, in turn, may trigger physiological changes, such as fluctuating hormone levels (29), metabolic dysregulation (30), and autonomic nervous system imbalance (31). These changes have the potential to increase the risk of cardiovascular disease (32), diabetes (33), and other chronic diseases (34, 35), thereby reducing life expectancy and increasing the risk of death. Other studies suggested that depressive symptoms may not only directly increase the risk of physical illness (36, 37) but may also be associated with the duration (38, 39) and severity (40, 41) of chronic disease, which, in turn, may lead to a higher risk of death in patients. These studies have shown an association between depressive symptoms and risk of death and chronic disease; but this association is unclear, and the findings are inconsistent. Therefore, the important relationship between depressive symptoms and health should be investigated and understood, especially the long-term relationship between depressive symptoms and all-cause mortality and chronic disease, which is crucial for effective management and alleviation of depressive symptoms and effective targeted treatment of depression.

Depression is a major public health problem in China (42, 43). The prevalence of depression among older people in China ranges from 27.0 to 37.3% (44, 45), which is much higher than that of the youth group between 5.3 and 17.5% (46, 47). Moreover, in recent years from 2015 to 2021, epidemiological studies have shown that middle-aged and older people are the most prevalent groups of depression (48), with different prevalence rates in different regions (49) and socioeconomic backgrounds (50). Numerous studies have found that compared to other depressed populations (e.g., adolescents), middle-aged adults have more persistent (51) and severe depressive symptoms (52), are more difficult to treat (53), and have depression for a longer period (54, 55). This means that most middle-aged adults with depressive symptoms are more likely to develop depression (7) and that, over time, depressive symptoms will have a greater impact on their physical health (52), leading to more serious physical illnesses (56), and may also coexist with chronic illnesses (57), which may worsen the progression of these illnesses (58, 59), increase the risk of self-inflicted suicide (60), and increase the risk of death (61). As China is about to enter an aging society, middle-aged and older people will account for an increasing proportion of the current and future Chinese population (62), with an estimated 500 million people over 80 by 2050 (63). Longitudinal studies, such as the China Health and Retirement Longitudinal Study (CHARLS), are needed to understand the long-term effects of depression and mortality in Chinese middle-aged and older people, specifically regarding exploring intrinsic mechanisms between depressive symptoms and all-cause mortality. Previous studies have also investigated the relationship between socioeconomic characteristics (64, 65) or lifestyle habits (66–68) and depression or mortality (69–71). For example, some studies have shown higher mortality rates in rural areas than in urban areas (72). However, a recent longitudinal study based on the *Abstract of Health Statistics* in Jiangxi Province, China, also found that middle-aged and older adults living in fast-paced cities had higher rates of depression and mortality than middle-aged and older adults living in natural rural environments (73). However, most of these multidimensional findings are from cross-sectional studies and lack long-term evidence of the adverse health effects of depression. Therefore, long-term follow-up studies are needed to explore the relationship between depressive symptoms and corresponding all-cause mortality in Chinese middle-aged and older people, considering socioeconomic characteristics and living habits.

More importantly, depressive symptoms frequently co-occur with various chronic diseases, such as cancer (74), diabetes (75), and cognition-related diseases (76), especially in older people (77, 78). Considering that chronic diseases are the most burdensome disease category among middle-aged and older people (79), highly incurring their mortality (80, 81), it is notably important to investigate the long-term relationships between depression and chronic diseases.

In conclusion, given the important impact of depression on the health of Chinese middle-aged and older people and the potential relationship between depressive symptoms and mortality in middle-aged and older people, it is necessary to conduct a longitudinal follow-up study in the middle-aged and older people to clarify the potential relationship between depressive symptoms and mortality as well as chronic diseases. Our objectives were (1) to explore the direct impact of depressive symptoms on subsequent mortality, (2) to map the relationship between depressive symptoms and chronic diseases and their effects on mortality, and (3) to compare the heterogeneity of such relationships in subgroups of different socioeconomic characteristics or living habits in Chinese middle-aged and older people.

## 2 Materials and methods

This study aimed to explore the association of depressive symptoms with all-cause mortality in middle-aged and older populations; data from CHARLS (2011, 2013, 2014, 2015, 2018 and 2020) was analyzed using Kaplan–Meier curves and Cox regression, with adjustments for covariates. The analysis included both overall and subgroup analyses. Overall analyses employed seven models to better observe the influence of different covariates on the outcome. Subgroup analyses consisted of a variety of three aspects, including socioeconomic variables, living habits, and patients with chronic diseases to analyze the association of depressive symptoms with all-cause mortality.

**Figure 1:**
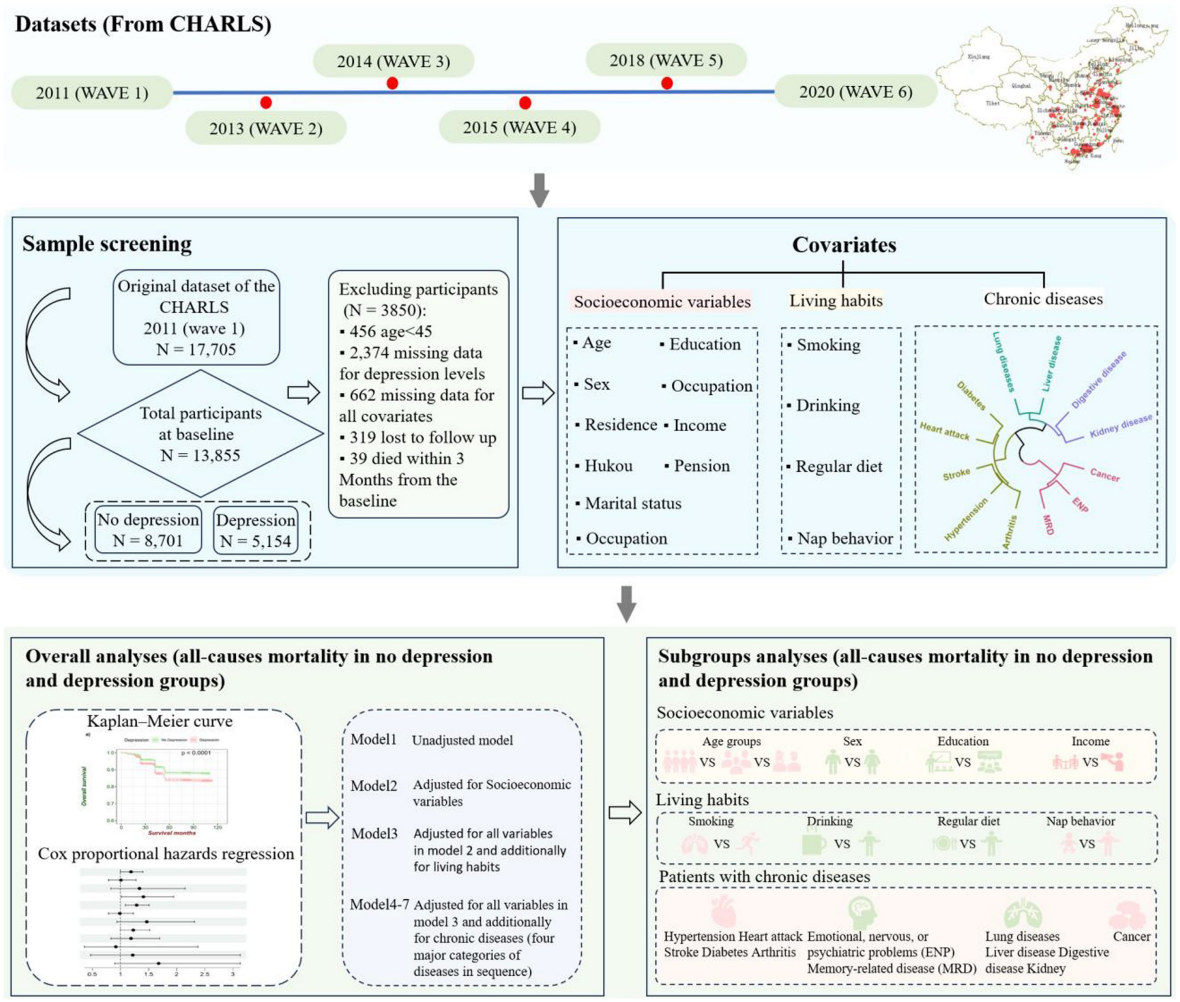
Schematic of the study workflow. First, data were obtained from the CHARLS database, and we used wave1(2011) as a baseline to get both the depressed and nondepressed populations as well as covariates and then tracked several rounds of data, including wave2 (2013), wave3 (2014), wave4 (2015), wave5 (2018), and wave6 (2020), to obtain survival information. Next, we selected appropriate covariates based on the research objectives and developed exclusion criteria for sample selection to obtain the necessary information for the study. After completing the data organization, we divided the all-cause mortality analysis of depressive symptoms in middle-aged and older people into overall analysis and subgroup analysis. In the overall analysis, we used the Kaplan–Meier curve and Cox proportional hazards regression to analyze whether depressive symptoms will increase the risk of all-cause mortality during the follow-up compared with individuals without depressive symptoms. To obtain more accurate results, we controlled for socioeconomic variables, lifestyle habits, and 12 chronic diseases, and established seven Cox proportional hazards regression models to explore the changes in hazard ratio (HR) values. Finally, we conducted subgroup analyses for socioeconomic variables, lifestyle habits, and various types of chronic disease patients separately, using the Kaplan–Meier curve and Cox proportional hazards regression.

The overall workflow chart is illustrated in Figure 1.

### 2.1 Study design, participants, and data collection

The data of this study comes from the CHARLS—a publicly accessible dataset upheld by the National School of Development at Peking University, explicitly concentrating on Chinese individuals aged 45 and above (82), which aims to collect a set of high-quality microdata representing households and individuals of middle-aged and older people aged 45 and above in China, which can be used to analyze the problem of aging population in China and to promote interdisciplinary research on aging (82). This database bears similarities to surveys like the Health and Retirement Survey (HRS) in the United States, the Elderly Follow-up Survey (ELSA) in the United Kingdom, and the European Health and Retirement Survey (SHARE). CHARLS conducted a countrywide baseline survey in 2011, followed by national surveys in 2013, 2014, 2015, 2018, and 2020, all of which can be accessed on the official website.^1^

In the current study, 10-year follow-up data was used from CHARLS—a population-based longitudinal cohort study of middle-aged and older people conducted in China (82). Details of the CHARLS study design and respondents were described elsewhere (82). In the baseline survey (wave 1), conducted between June 2011 and March 2012, 17,705 participants were recruited (82). After the baseline interview, subsequent three follow-ups were conducted in 2013 (wave 2), 2014 (wave 3), and 2015 (wave4), 2018 (wave5), and 2020 (wave6), respectively (82). CHARLS was approved by the Institutional Review Board of Peking University and all methods were performed by the relevant guidelines and regulations. All respondents provided written informed consent. If the respondent was illiterate, he/she would press the fingerprint after the interviewer who dictated the content of the informed consent. According to analytical purposes, we excluded participants with the following criteria: (1) individuals under 45 years at baseline (*n* = 456), (2) individuals without information of all variables (Including the measured depressive symptom levels and all covariates) at baseline (*n* = 3,036), (3) individuals who failed to follow up (*n* = 319), (4) individuals who died within 3 months from the baseline (*n* = 39) (83, 84). Finally, 13,855 participants were included in the analysis (Figure 1). Initially, our research necessitated the examination of the association between depression symptoms and all-cause mortality in middle-aged and older people over the age of 45, so the baseline population under 45 years old was excluded. Subsequently, any missing data were eliminated for all variables at baseline to obtain complete data information. To obtain a more accurate estimate of survival time, participants who lost to follow-up were excluded. Finally, considering the bias in mortality statistics, the number of deaths within 3 months from the baseline was excluded.

### 2.2 Variables

#### 2.2.1 Psychopathological symptom measures for depressive symptoms

The study measured depressive symptoms in older Chinese respondents using the Center for Epidemiologic Studies Depression Scale (CES-D-10), and the reliability and validity are well-supported by extensive research (85–87), with Cronbach’s alpha values being greater than 0.8, which was validated through the CHARLS. The CES-D-10 questionnaire consists of 10 items that assess how the participant felt and behaved during the past week. Each item contains four response options: (1) rarely or none of the time (< 1 day); (2) some or a little of the time (1–2 days); (3) occasionally or a moderate amount of the time (3–4 days); and (4) most or all the time (5–7 days). The four response options are scored on a scale from 0 to 3, and the total scores of CES-D-10 range from 0 to 30. Higher scores indicate more severe depressive symptoms. A cutoff score of ≥10 was used to identify participants with serious depressive symptoms in this study. Scores were defined as follows: 0–9 points indicated no depressive symptoms, while a score of 10–30 indicated depressive symptoms. The CES-D-10 demonstrated solid internal consistency in the 2011 survey, with a Cronbach’s alpha of 0.82.

#### 2.2.2 Covariates for socioeconomic characteristics, living habits, and chronic disease

Covariates were defined from the baseline survey. In the current study, age groups, sex, Hukou (a household registration record officially identifies a person as a permanent resident of an area and includes identifying information such as name, parents, spouse, and date of birth), marital status, education, income, pension, occupation, smoking, drinking, regular diet and nap behavior, and self-reported history of chronic diseases, which were included to the overall analyses of the relationship between depressive symptoms and all-cause mortality.

Age groups were categorized as 45–54 years, 55–64 years, and more than 65 years. Residence was categorized as rural and urban. Hukou was categorized as agricultural and nonagricultural. Marital status was classified into living without a spouse and living with a spouse. Education status was dichotomized as illiterate versus literate. Income levels were categorized as below-median (1,960 yuan) and above-median household income. Pension status was categorized as no pension and pension. Smoking status was dichotomized as ever versus never and drinking status as ever versus never. Regular diet was categorized as No (not 3 meals per day) and Yes (3 meals per day), and nap behavior was classified into No and Yes.

Chronic diseases were measured from the item “Have you been diagnosed with [conditions listed below, read one by one] by a doctor?,” and all dichotomized as Yes versus No, which included diseases of the circulatory system, the endocrine and the musculoskeletal system (hypertension, heart attack, stroke, diabetes, and arthritis), diseases of the respiratory system, the digestive system and the urinary system (lung diseases, liver disease, digestive disease, and kidney), neoplasms (cancer), and mental, behavioral, or neurodevelopmental disorders (emotional, nervous, or psychiatric problems and memory-related disease).

### 2.3 Assessment of mortality

Mortality was determined by the interview status (alive or dead) of participants in waves 2, 3, 4, 5, and 6. The information on the interview date could be obtained from all three follow-ups, but the exact death time was only available in Wave 2 and Wave 6. If death events occurred with exact records, the survival time was calculated as the interval from the date of the baseline survey to the date of the participant’s death. If no death data is available, the survival time was estimated based on the median time from the date of the first interview to the wave with the death record.

## 3 Statistical analysis

First, data was obtained from the CHARLS database, and we used wave1(2011) as a baseline to get both the depressed and nondepressed groups as well as covariates and then tracked several rounds of data, including wave2 (2013), wave3 (2014), wave4 (2015), wave5 (2018), and wave6 (2020), to obtain survival information. Next, we selected appropriate covariates based on the research objectives and developed exclusion criteria for sample selection to obtain the necessary information for the study. After completing the data organization, we divided the all-cause mortality analysis of depressive symptoms in middle-aged and older people into overall analysis and subgroup analysis. In the overall analysis, we used the Kaplan–Meier curve and Cox proportional hazards regression to analyze whether depressive symptoms will increase the risk of all-cause mortality during the follow-up compared with individuals without depressive symptoms. To obtain more accurate results, we controlled for socioeconomic variables, lifestyle habits, and 12 kinds of chronic diseases, and established seven Cox proportional hazards regression models to explore the changes in hazard ratio (HR) values. Finally, we conducted subgroup analyses for socioeconomic variables, lifestyle habits, and various types of chronic disease patients with Kaplan–Meier curve and Cox proportional hazards regression separately.

### 3.1 Overall analyses of the relationship between depressive symptoms and all-cause mortality

Before overall analyses of the relationship between depressive symptoms and all-cause mortality, it needs to test the difference in the categorical and continuous variables between the depressive symptoms group and the no depressive symptoms groups. Categorical variables were presented as count (percentage), and the differences in baseline characteristics across the depressive symptom levels were compared by the Pearson Chi-square test. As for the continuous variable (only age), it was needed to conduct a normality test to determine whether to use parametric or nonparametric tests to assess the difference in the age between the depressive symptoms group and the no depressive symptoms group. Since our sample size was greater than 5,000, the Kolmogorov–Smirnov test was used for the normality test. The test results showed that age is skewed (*p* < 0.001), so the difference in age between the depressive symptoms group and the no depressive symptoms group was assessed using the nonparametric Mann–Whitney U test, and age was expressed as median (*Q*1, *Q*3).

The methods of the Kaplan–Meier curve and Cox proportional hazards regression were utilized to analyze the overall relationship between depressive symptoms and all-cause mortality. The Kaplan–Meier curve was used to show the incident rates of mortality by depressive symptom levels and Cox proportional hazards regression models were used to explore the association between depressive symptoms and all-cause mortality with hazard ratios (HRs) and 95% confidence intervals (CIs). In the Cox proportional hazards regression models, the endpoint event was death, and the censored event was designated to survive. To better observe the influence of different covariates on the outcome, 7 Cox proportional hazards regression models were constructed. Model 1 referred to the unadjusted model. Model 2 was adjusted for all variables in model 1 and additionally for age groups, sex, Hukou, marital status, education, income, pension, and occupation. Model 3 was adjusted for all variables in model 2 and additionally for smoking, drinking, regular diet, and nap behaviors. Model 4 was adjusted for all variables in model 3 and additionally for hypertension, heart attack, stroke, diabetes, and arthritis. Model 5 was adjusted for all variables in model 4 and additionally for lung, liver, digestive, and kidney diseases. Model 6 was adjusted for all variables in model 5 and additionally for cancer. Model 7 was adjusted for all variables in model 6 and, additionally, for emotional, nervous, or psychiatric problems (ENP) and memory-related disease (MRD).

### 3.2 Subgroup analyses of the relationship between depressive symptoms and all-cause mortality

Subgroup analyses consisted of varieties of three aspects, including socioeconomic variables, living habits, and patients with chronic diseases to analyze the association of depressive symptoms with all-cause mortality.

#### 3.2.1 The association between socioeconomic characteristics or living habits and depressive symptoms

Also, the Kaplan–Meier curve was used to show the incident rates of mortality by depressive symptom levels, and Cox proportional hazards regression models were used to explore the association between depressive symptoms and all-cause mortality with hazard ratios (HRs) and 95% confidence intervals (CIs) in such subgroup analysis.

Subgroup analysis of socioeconomic characteristics was carried out by different age groups (45–54 years old, 55–64 years old, and ≥ 65 years old), sex (male and female), residence (rural and urban), Hukou (agricultural and nonagricultural), marital status (living with spouse and living without spouse), education (illiterate and literate), income (below median and above median), pension (pension and no pension), occupation (agricultural work and nonagricultural work). And subgroup analysis of living habits was carried out by smoking (No and Yes), drinking (No and Yes), regular diet (No and Yes), and nap behavior (No and Yes).

#### 3.2.2 The relationship between depressive symptoms and chronic diseases and their effect on mortality

To directly ascertain the association between depressive symptoms and chronic diseases and their effect on mortality, Cox proportional hazards regression models were only used to explore the association between depressive symptoms and all-cause mortality with hazard ratios (HRs) and 95% confidence intervals (CIs), controlling for age groups, sex, Hukou, marital status, education, income, pension, occupation, smoking, drinking, and regular diet and nap behaviors.

Diseases such as circulatory system, endocrine, and musculoskeletal system diseases (hypertension, heart attack, stroke, diabetes, and arthritis), respiratory system, digestive system, and urinary system diseases (lung diseases, liver disease, digestive disease, and kidney), neoplasms (cancer), and mental, behavioral, or neurodevelopmental disorders (emotional, nervous, or psychiatric problems and memory-related disease) were analyzed to understand whether depressive symptoms increase the risk of all-cause mortality in patients with chronic diseases.

All statistical analyses were conducted with *R* 4.2.1 and a two-sided *p*-value.

## 4 Results

### 4.1 Descriptive statistics of participants in cohort description

From a total of 13,855 participants, 8,701 individuals were without depressive symptoms (62.8%) and 5,154 individuals were with depressive symptoms (37.2%), as seen in Table 1. Significant differences in socioeconomic variables, living habits, and chronic diseases were found between middle-aged and older people with depressive symptoms and those without. Older females who lived in rural areas and had an agricultural household were more likely to have depressive symptoms. Specifically, the median age of middle-aged and older people with depressive symptoms was 59 years (53.00%, 66.00%), whereas the median age of the population without depressive symptoms was 57 years (51.00%, 64.00%). Among those with depressive symptoms, the proportion of middle-aged and older females was 60.40%, compared with 46.56% in the group without depressive symptoms. In addition, among middle-aged and older people with chronic diseases, such as high blood pressure, digestive diseases, and arthritis, the proportion of those with depressive symptoms was 27.47%, 30.56%, and 45.87%, respectively, while the proportion of those without depressive symptoms was 22.97%, 17.47%, and 26.12%. These data show an association between depressive symptoms and several factors.

**Table 1:** Baseline characteristics of the study population.

Variables	No depressive symptoms	Depressive symptoms	χ^2^ or W-value	*p*-value
No. of participants	8,701	5,154		
Age [median (*Q*1, *Q*3)]	57.00 (51.00, 64.00)	59.00 (53.00, 66.00)	20098288.50	<0.001
Age groups, *n* (%)			90.24	<0.001
45–54	3,391 (38.97)	1,619 (31.41)		
55–64	3,209 (36.88)	2018 (39.15)		
≥65	2,101 (24.15)	1,517 (29.43)		
Sex, *n* (%)			247.80	<0.001
Male	4,650 (53.44)	2041 (39.60)		
Female	4,051 (46.56)	3,113 (60.40)		
Residence, *n* (%)			219.45	<0.001
Rural	4,879 (56.07)	3,546 (68.80)		
Urban	3,822 (43.93)	1,608 (31.20)		
Hukou, *n* (%)			244.34	<0.001
Agricultural	6,424 (73.83)	4,392 (85.22)		
Nonagricultural	2,277 (26.17)	762 (14.78)		
Marital status, *n* (%)			149.87	<0.001
Living without spouse	1,197 (13.76)	1,124 (21.81)		
Living with spouse	7,504 (86.24)	4,030 (78.19)		
Education, *n* (%)			242.53	<0.001
Illiterate	1924 (22.11)	1764 (34.23)		
Literate	6,777 (77.89)	3,390 (65.77)		
Income, *n* (%)			14.00	<0.001
Below median	4,383 (50.37)	2,426 (47.07)		
Above median	4,318 (49.63)	2,728 (52.93)		
Pension, *n* (%)			63.32	<0.001
No pension	6,744 (77.51)	4,286 (83.16)		
Pension	1957 (22.49)	868 (16.84)		
Occupation, *n* (%)			34.70	<0.001
Agricultural work	4,627 (53.18)	3,007 (58.34)		
Nonagricultural work	4,074 (46.82)	2,147 (41.66)		
Smoking, *n* (%)			55.49	<0.001
No	5,022 (57.72)	3,306 (64.14)		
Yes	3,679 (42.28)	1848 (35.86)		
Drinking, *n* (%)			125.60	<0.001
No	5,543 (63.71)	3,761 (72.97)		
Yes	3,158 (36.29)	1,393 (27.03)		
Regular diet, *n* (%)			133.10	<0.001
No	1,094 (12.57)	1,025 (19.89)		
Yes	7,607 (87.43)	4,129 (80.11)		
Nap behavior, *n* (%)			60.52	<0.001
No	3,845 (44.19)	2,630 (51.03)		
Yes	4,856 (55.81)	2,524 (48.97)		
Hypertension, *n* (%)			35.04	<0.001
No	6,702 (77.03)	3,738 (72.53)		
Yes	1999 (22.97)	1,416 (27.47)		
Heart attack, *n* (%)			178.11	<0.001
No	7,883 (90.6)	4,272 (82.89)		
Yes	818 (9.4)	882 (17.11)		
Stroke, *n* (%)			52.65	<0.001
No	8,570 (98.49)	4,979 (96.6)		
Yes	131 (1.51)	175 (3.4)		
Diabetes, *n* (%)			17.63	<0.001
No	8,256 (94.89)	4,801 (93.15)		
Yes	445 (5.11)	353 (6.85)		
Arthritis, *n* (%)			565.76	<0.001
No	6,428 (73.88)	2,790 (54.13)		
Yes	2,273 (26.12)	2,364 (45.87)		
Lung diseases, *n* (%)			143.68	<0.001
No	8,016 (92.13)	4,418 (85.72)		
Yes	685 (7.87)	736 (14.28)		
Liver disease, *n* (%)			28.38	<0.001
No	8,417 (96.74)	4,891 (94.9)		
Yes	284 (3.26)	263 (5.1)		
Digestive disease, *n* (%)			318.91	<0.001
No	7,181 (82.53)	3,579 (69.44)		
Yes	1,520 (17.47)	1,575 (30.56)		
Kidney disease, *n* (%)			125.76	<0.001
No	8,299 (95.38)	4,666 (90.53)		
Yes	402 (4.62)	488 (9.47)		
Cancer, *n* (%)			4.51	0.034
No	8,635 (99.24)	5,096 (98.87)		
Yes	66 (0.76)	58 (1.13)		
ENP, *n* (%)			77.98	<0.001
No	8,657 (99.49)	5,043 (97.85)		
Yes	44 (0.51)	111 (2.15)		
MRD, *n* (%)			29.82	<0.001
No	8,630 (99.18)	5,057 (98.12)		
Yes	71 (0.82)	97 (1.88)		

ENP, emotional, nervous, or psychiatric problems; MRD, memory-related disease.

### 4.2 Overall analyses of the relationship between depressive symptoms and all-cause mortality

**Figure 2:**
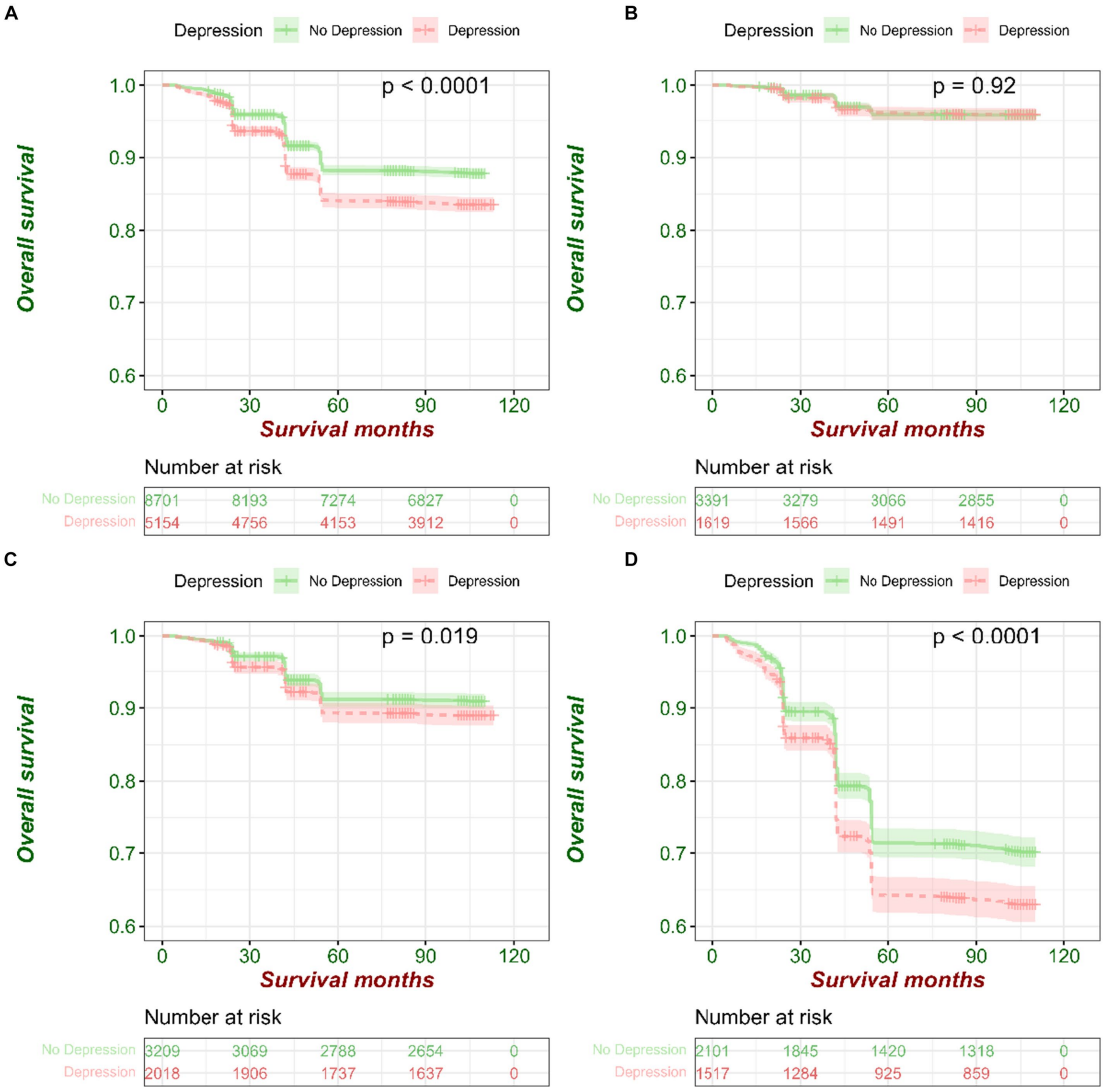
The Survival curves by depressive symptom levels in middle-aged and older people from 2011 to 2020. **(A)** Survival curves by depressive symptom levels in all samples. **(B)** Survival curves by depressive symptom levels in aged 45–54 years group. **(C)** Survival curves by depressive symptom levels in aged 55–64 years group. **(D)** Survival curves by depressive symptom levels in aged 65 years and above group.

During the 10-year follow-up, 1862 death events were reported, with a crude mortality rate of 13.44 per 1,000 person-years. Individuals with depressive symptoms possessed a higher mortality rate than those without depressive symptoms (crude mortality rates, 16.12 and 11.85 per 1,000 person-years, respectively; Figure 2A; Supplementary eFigure 2). The unadjusted risk estimates (model 1) showed that individuals with depressive symptoms had a 40% higher risk of all-cause mortality during the follow-up compared with individuals without depressive symptoms (HR = 1.40 [95% CI, 1.28–1.53]; Figure 2). This risk remained significant when adjusting for age groups, sex, Hukou, marital status, education, income, pension, and occupation in model 2 (HR = 1.30 [95% CI, 1.18–1.42]; Supplementary eFigure 2). Model 3 adjusted for all variables in model 2 and additionally for smoking, drinking, regular diet, and nap behavior showed significantly higher risks for all causes of death (HR = 1.28 [95% CI, 1.16–1.40]; Supplementary eFigure 2). Model 4 to model 7 adjusted for all variables in model 3 and additionally for chronic diseases (four major categories of diseases in sequence) showed significantly higher risks for all causes of death (model 4: HR = 1.23 [95% CI, 1.12–1.36]; model 5: HR = 1.21 [95% CI, 1.09–1.33]; model 6: HR = 1.21 [95% CI, 1.09–1.33]; and model 7: HR = 1.20 [95% CI, 1.09–1.33]; Supplementary eFigure 2).

### 4.3 Subgroup analyses of the relationship between depressive symptoms and all-cause mortality

#### 4.3.1 The association between socioeconomic characteristics or living habits and depressive symptoms

**Figure 3:**
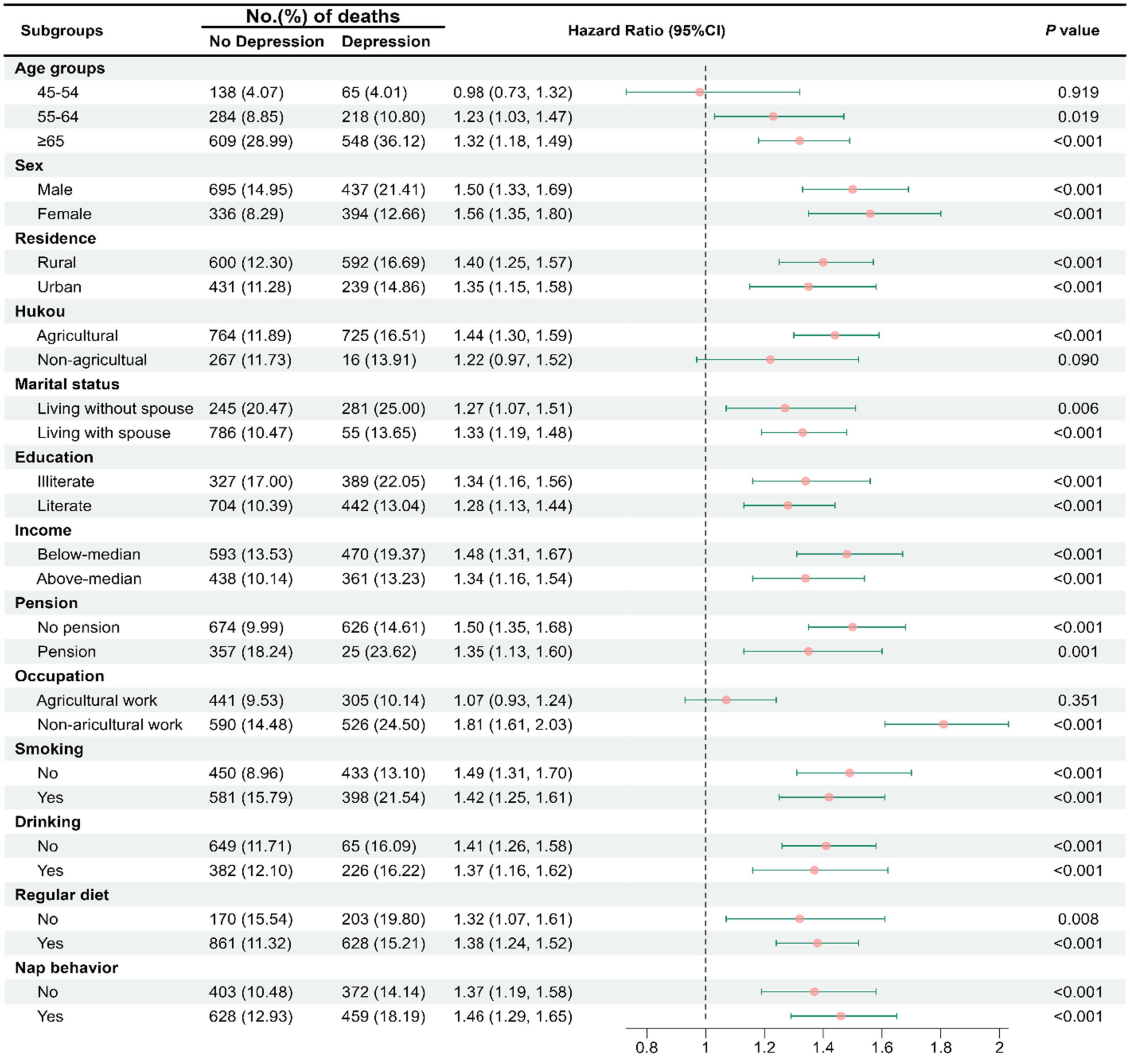
Subgroup analyses of socioeconomic characteristics or living habits in the association of depressive symptoms with all-cause mortality.

The results of subgroup analysis (Figure 3) show that for the results of age groups (45–54 years, 55–64 years, and ≥ 65 years), Hukou (agricultural and nonagricultural), occupations (agricultural work and nonagricultural work), the impact of depressive symptoms on all-cause mortality only occurred in those who were in 55–64 years old (HR = 1.23 [95% CI, 1.03–1.47]; Figures 2C, 3), more than 65 years old (HR = 1.32 [95% CI, 1.18–1.49]; Figures 2D, 3), agricultural Hukou (HR = 1.44 [95% CI, 1.30–1.59]; Supplementary eFigure 1A; Figure 3), and nonagricultural work (HR = 1.81 [95% CI, 1.61–2.03]; Supplementary eFigure 1D; Figure 3).

#### 4.3.2 The relationship between depressive symptoms and patients with chronic diseases and their effect on mortality patients with chronic diseases

**Figure 4:**
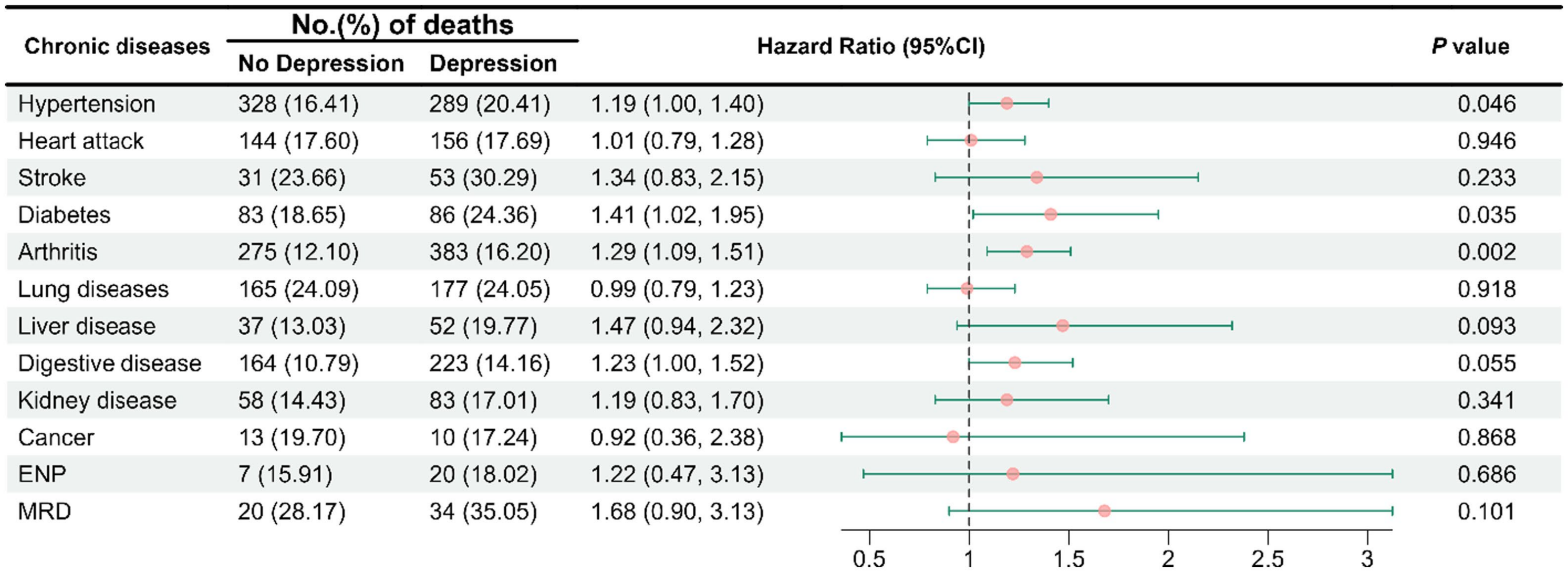
Hazard ratios for all-cause mortality among individuals with chronic diseases with depressive symptom levels. The model was adjusted for age groups, sex, Hukou, marital status, education, income, pension, occupation, smoking, drinking, regular diet, and nap behavior. ENP, emotional, nervous, or psychiatric problems; MRD, memory-related disease.

Analyses controlling for age groups, sex, Hukou, marital status, education, income, pension, occupation, smoking, drinking, regular diet, and nap behaviors, the results suggest depressive symptoms increased the risk of all-cause mortality among patients with hypertension (HR = 1.19 [95% CI, 1.00–1.40]), diabetes (HR = 1.41 [95% CI, 1.02–1.95]), and arthritis (HR = 1.29 [95% CI, 1.09–1.51]; Figure 4).

## 5 Discussion

To the best of our knowledge, this is the first longitudinal study in China to focus on the causes of mortality in middle-aged and older people with depressive symptoms. The result of the overall analyses of the relationship between depressive symptoms and all-cause mortality suggests that middle-aged and older individuals with depressive symptoms have a higher all-cause mortality rate, showing a 20% increased risk compared to those without depressive symptoms. In addition, the risk of mortality among middle-aged and older people with depressive symptoms increases significantly with age, and depressive symptoms exert a more pronounced influence on mortality risk among urban residents with heavy workloads and rural residents. Our findings also indicate that co-occurring depressive symptoms in middle-aged and older people with hypertension, diabetes, or arthritis increased the risk of mortality. All these findings indicate that depressive symptoms can significantly increase the risk of mortality, underlining the lethality of depressive symptoms for middle-aged and older people. Our findings provide innovative insights for developing effective intervention policies to slow down the surging middle-aged and older people mortality accelerated by depressive symptoms.

An expanding body of research has consistently shown the significant impact of depressive symptoms on individuals’ mental and physical well-being (88, 89). Our study further controlled for socioeconomic variables, lifestyle factors, and known chronic diseases associated with life expectancy. Through a decade-long follow-up, we found a highly significant association between depressive symptoms and all-cause mortality, implying that depressive symptoms have a significant negative impact on shortening life expectancy and increasing the risk of death.

The increase in mortality among older people due to depressive symptoms becomes more pronounced with different ages. People with depressive symptoms in the 55–64 and 65-and-over age brackets exhibit significantly higher overall mortality risks compared to their nondepressed counterparts aged 45–54 years. Our findings align with numerous previous cross-sectional research results, suggesting that the impact of depressive symptoms on mortality risk in middle-aged adults might be manageable, while the lethal effects of depressive symptoms in older people might be irreversible. Middle-aged individuals are typically in better health (90) as they seek for medical help from themselves or others (91), follow medical advice (92), and receive progressive treatment (93). On the contrary, older adults may experience poorer treatment outcomes and a higher risk of death from depressive symptoms as aging may impair their perception (94), cognitive abilities (95), verbal expression (96), comprehension (97), help-seeking ability (98), and sabotage treatment collaboration and compliance (99).

Following subgroup analyses based on household registration and occupation, the impact of depressive symptoms on the overall mortality rate was observed in the rural population and among the labor force with nonagricultural urban occupations. Our longitudinal research results correspond with previous research on how socioeconomic factors influence individuals with depressive symptoms (100), indicating that over an extended period, individuals with depressive symptoms living in rural areas (84) and those not engaged in agricultural work experience share higher mortality rates (101). This underscores the lasting impact of poorer socioeconomic factors on reducing survival rates for individuals with depressive symptoms (102). Rural areas, compared to urban settings, often show a significant deficiency in mental health services (103). The scarcity of professionals and facilities may lead to depressive symptoms not being timely and effectively identified and treated (104, 105). Moreover, the middle-aged and older population in rural areas may have more fragile social support relative to their urban counterparts (106). A lack of social support may make the impact of depressive symptoms on mortality rates more pronounced, as social support is a crucial factor in alleviating symptoms of depressive symptoms and improving mental health (107). Therefore, this emphasizes the necessity of prioritizing medical care and support conditions for depressed individuals in rural areas to potentially extend their lifespan. Additionally, depressed individuals not engaged in agricultural work face a higher risk of death, suggesting that labor work and exercise activity may have positive effects on reducing depression. Individuals engaged in nonagricultural occupations may be more susceptible to unhealthy lifestyle choices such as smoking, alcohol consumption, and lack of physical activity (108–110). These lifestyle factors can interact with symptoms of depression, collectively increasing the risk of mortality (109, 111). Furthermore, nonagricultural work is often associated with heightened job stress and competition, which may exacerbate the development of depressive symptoms (112). The long-term interplay between work-related stress and depressive symptoms can lead to health issues such as cardiovascular diseases, thereby affecting mortality rates (113). However, the authenticity of the effects requires further investigation.

In addition, our findings also indicate that middle-aged and older people with hypertension, diabetes, or arthritis who also suffer from depressive symptoms are at a higher risk of mortality. Numerous studies have already demonstrated the association between depressive symptoms and cardiovascular diseases, diabetes, chronic inflammation, and immune system disorders (113–116). These cross-sectional studies have indicated that depressive symptoms exacerbate the condition of chronic diseases. Our 10-year longitudinal study affirms that depressive symptoms significantly magnify the impact of hypertension, diabetes, and arthritis on reducing the life expectancy of middle-aged and older people, and this impact becomes apparent within a decade. Depressive symptoms diminish middle-aged and older individuals’ emotional regulation (117) and resilience to stress (118), heightening the challenge of managing mood and blood pressure for those with hypertension (119). Patients with depressive symptoms often exhibit an imbalance in the autonomic nervous system, particularly an overactivation of the sympathetic nervous system (120). This activation may lead to increased heart rates and vasoconstriction, thereby elevating cardiac output and peripheral vascular resistance, ultimately resulting in heightened blood pressure (121). Additionally, depressive symptoms can lead to hyperactivity of the hypothalamic–pituitary–adrenal (HPA) axis (122), which may cause elevated levels of cortisol (123). Sustained high levels of cortisol can disrupt blood pressure regulation mechanisms (124), increasing the risk of hypertension (125). For individuals with diabetes, depressive symptoms (126) may increase the likelihood of severe lethal complications (127, 128), especially the risk of death from diabetes complications such as stroke (129) and heart failure (130). Individuals with depressive symptoms may also present stress responses and inflammatory reactions, leading to the development of insulin resistance syndrome, which includes abdominal fat deposition, elevated plasma triglycerides, and insulin levels risk factors for type 2 diabetes (131–133). Additionally, people with depressive symptoms may have a chronic low-grade inflammatory state, with elevated levels of inflammatory markers such as tumor necrosis factor-alpha (TNF-α) and interleukin-6 (IL-6) (134–136). These inflammatory markers not only affect mood and behavior but may also cause joint damage (137), promoting the onset and progression of arthritis (138), thus increasing the risk of arthritis (139). These factors collectively elevate the mortality risk caused by chronic conditions.

The study provides a comprehensive longitudinal perspective, revealing that depressive symptoms not only significantly shorten the survival time and increase the risk of death, but they have also been shown to act synergistically with chronic diseases such as hypertension, diabetes, and arthritis, further amplifying the overall risk of death among middle-aged and older adults. It should be further clarified that long-term depressive symptoms may not only lead to the development of chronic diseases in middle-aged and older people but also increase the risk of death from chronic diseases in middle-aged and older people. At the same time, those who are already chronically ill may also develop persistent and severe depressive symptoms over time, which may increase the burden of chronic disease on their health and further increase the risk of death in older people. The research findings underscore the lethality of depressive symptoms among the middle-aged and older population, highlighting the need for healthcare professionals to enhance early identification and intervention for depressive symptoms in clinical practice. Regular mental health assessments and screenings should be conducted to promptly detect depressive symptoms and provide patients with necessary psychological support and treatment. Concurrently, considering the synergistic effects of depressive symptoms with other chronic conditions, clinical treatment plans should be more comprehensive, focusing not only on the treatment of depression itself but also on the overall health status of the patient, including the management of chronic diseases such as hypertension, diabetes, and arthritis. Public health departments should raise public awareness of depressive symptoms and their association with other chronic diseases through education and outreach campaigns, encouraging middle-aged and older individuals to actively seek help and dispel prejudices and stigma surrounding mental health issues (140). Additionally, governments should enact relevant policies to provide financial support and resource guarantees for mental health services for the middle-aged and older, ensuring that all middle-aged and older individuals have access to necessary mental health care (141). On an individual level, it is recommended that middle-aged and older individuals sign up for comprehensive health checks regularly, including mental health assessments, to facilitate the timely detection and management of depressive symptoms and other chronic conditions (142). Living a healthy lifestyle, such as regular physical exercise, a balanced diet, and good sleep habits, can reduce the risk of chronic diseases and improve mental health (143). Furthermore, family members’ and community’s participation in the mental health support of middle-aged and older people should be encouraged, offering emotional support and assistance in daily life to strengthen their social connections and sense of belonging (144).

## 6 Strengths and limitations

This study has several strengths. It benefits from the nationwide cohort design, with nearly 10-year follow-up, and meticulous adjustments for crucial socioeconomic variables, living habits, and comorbid chronic conditions. Furthermore, to our knowledge, this cohort is probably one of the most representative studies of older people in China with depression conducted to date.

This study also has several limitations. Firstly, as the CES-D-10 depression scale used is not a diagnostic criterion for depression, the scale responds more to the level of depressive symptoms over a certain period and is used for the assessment of the severity of depressive symptoms already for the rapid classification of potential patients with depression (145). Therefore, it should be recognized in all studies using the CES-D-10 as a depression assessment tool that the CES-D-10 is a measure of the level of depressive symptoms, and that higher scores on the scale respond to greater severity of depressive symptoms, but are not diagnostic of depression (146). The fact that there is not a direct causal relationship between depressive symptoms and depression makes it clear that more in-depth research is needed to examine the relationship between depressive symptoms and depression, as well as mortality and chronic disease. Additionally, there may be cases of underdiagnosis in the screening of depression, which could lead to bias as the CES-D-10 depression scale used is a self-assessment tool. Middle-aged and older people may incorrectly fill it out due to the stigma associated with depression diagnosis. This implies that some middle-aged and older people with depressive symptoms may not have been included in the cohort, making the estimation results unclear to a certain extent. It is also important to note that depression score data were recorded only at baseline. Depression scores may change with age, which could affect study results. Also, as a result, our study is unable to determine the specific dynamic process of the effect of changes in depressive symptoms over time on the risk of death; therefore, prospective cohort studies or randomized controlled trials may be needed in future studies to further validate the potential dynamic developmental changes between changes in depressive symptoms over time and the risk of death. Thirdly, although many potential confounding variables were adjusted for, it is still possible that some residual confounding factors, such as the endogeneity between lifestyle factors (such as smoking or drinking) and depressive symptoms, may have hindered the identification of stronger associations. Additionally, the CHARLS dataset did not provide information on cause-specific mortality, which prevented investigation into the predictive value of depressive symptoms for specific causes of death. Variables like these should be included in future follow-up studies to help researchers expand on current findings among the Chinese population. Finally, it is required to explore the potential mechanisms or pathways by which depressive symptoms may lead to an increase in all-cause mortality in Chinese middle-aged and older people in the future to reduce the risk of death due to depression. In this study, the findings identified an association between depressive symptoms and all-cause mortality in acute and chronic diseases in middle-aged and older people, suggesting that intervention and treatment of depression should focus on the early stage of depressive symptom emergence. The potential mechanisms and pathways of this association should be further explored in future studies to reduce the risk of death due to depression in middle-aged and older people in China.

## 7 Conclusion

In this cohort study, depressive symptoms are associated with an increased risk of all-cause mortality, especially among the rural household population aged 55 years and older and among those who worked in nonagricultural jobs. The findings suggest that not only are depressive symptoms strongly associated with an increased risk of all-cause mortality among Chinese middle-aged and older adults but also that when depressive symptoms are comorbid with chronic disease, depressive symptoms increase the risk of all-cause mortality among middle-aged and older adults with chronic disease. The findings suggest that interventions such as early detection, integrated chronic disease care management, healthy lifestyles, and community support for depressive symptoms may help to buffer against mortality among middle-aged and older adults from the perspective of interventions for depression in middle-aged and older adults.

## Data availability statement

The original contributions presented in the study are included in the article/Supplementary material, further inquiries can be directed to the corresponding author.

## Ethics statement

The studies involving human participants were reviewed and approved by the Center for Healthy Aging and Development Studies at Peking University and Duke University. The studies were conducted by the local legislation and institutional requirements. The participants provided their written informed consent to participate in this study. YW registered the platform and obtained permission to use the data.

## Author contributions

LZ: Conceptualization, Formal analysis, Investigation, Methodology, Writing – original draft, Writing – review & editing. YiW: Investigation, Methodology, Writing – review & editing. JL: Conceptualization, Software, Supervision, Visualization, Writing – review & editing. HZ: Formal analysis, Funding acquisition, Methodology, Software, Writing – review & editing. NL: Resources, Validation, Writing – review & editing. YuW: Project administration, Resources, Supervision, Validation, Writing – review & editing.

## Footnotes

^1^ http://charls.ccer.edu.cn/zh-CN

## References

[1] Ostergaard SD Rothschild AJ Uggerby P Munk-Jørgensen P Bech P Mors O. Considerations on the ICD-11 classification of psychotic depression. Psychother Psychosom. (2012) 81:135–44. doi: 10.1159/000334487, PMID: 22398817

[2] Broomhall AG Phillips WJ Hine DW Loi NM. Upward counterfactual thinking and depression: a meta-analysis. Clin Psychol Rev. (2017) 55:56–73. doi: 10.1016/j.cpr.2017.04.010, PMID: 28501706

[3] Okereke OI Reynolds CF 3rd Mischoulon D Chang G Vyas CM Cook NR. Effect of Long-term vitamin D3 supplementation vs placebo on risk of depression or clinically relevant depressive symptoms and on change in mood scores: a randomized clinical trial. JAMA. (2020) 324:471–80. doi: 10.1001/jama.2020.10224, PMID: 32749491 PMC7403921

[4] Price RB Duman R. Neuroplasticity in cognitive and psychological mechanisms of depression: an integrative model. Mol Psychiatry. (2020) 25:530–43. doi: 10.1038/s41380-019-0615-x, PMID: 31801966 PMC7047599

[5] Deng J Zhou F Hou W Silver Z Wong CY Chang O. The prevalence of depressive symptoms, anxiety symptoms and sleep disturbance in higher education students during the COVID-19 pandemic: a systematic review and meta-analysis. Psychiatry Res. (2021) 301:113863. doi: 10.1016/j.psychres.2021.113863, PMID: 33984824 PMC9225824

[6] Reich A Weiß A Lindner L Baraliakos X Poddubnyy D Zinke S. Depressive symptoms are associated with fatigue, poorer functional status and less engagement in sports in axSpA and PsA: an analysis from the RABBIT-SpA cohort. Arthritis Res Ther. (2023) 25:136. doi: 10.1186/s13075-023-03127-2, PMID: 37533077 PMC10394807

[7] Rice F Riglin L Lomax T Souter E Potter R Smith DJ. Adolescent and adult differences in major depression symptom profiles. J Affect Disord. (2019) 243:175–81. doi: 10.1016/j.jad.2018.09.015 30243197

[8] Park KE Lee H Kwon YD Kim S. Association between changes in Oral health-related quality of life and depressive symptoms in the Korean elderly population. Int J Public Health. (2023) 68:1605403. doi: 10.3389/ijph.2023.1605403, PMID: 37065644 PMC10103146

[9] Shorey S Ng ED Wong C. Global prevalence of depression and elevated depressive symptoms among adolescents: a systematic review and meta-analysis. Br J Clin Psychol. (2022) 61:287–305. doi: 10.1111/bjc.12333, PMID: 34569066

[10] Eckshtain D Marchette LK Schleider J Weisz JR. Parental depressive symptoms as a predictor of outcome in the treatment of child depression. J Abnorm Child Psychol. (2018) 46:825–37. doi: 10.1007/s10802-017-0323-4, PMID: 28643207 PMC5742091

[11] Valtonen HM Suominen K Mantere O Leppämäki S Arvilommi P Isometsä ET. Prospective study of risk factors for attempted suicide among patients with bipolar disorder. Bipolar Disord. (2006) 8:576–85. doi: 10.1111/j.1399-5618.2006.00341.x 17042831

[12] Laursen TM Musliner KL Benros ME Vestergaard M Munk-Olsen T. Mortality and life expectancy in persons with severe unipolar depression. J Affect Disord. (2016) 193:203–7. doi: 10.1016/j.jad.2015.12.067, PMID: 26773921

[13] Santomauro DF Herrera A Shadid J Zheng P Ashbaugh C Pigott DM. Global prevalence and burden of depressive and anxiety disorders in 204 countries and territories in 2020 due to the COVID-19 pandemic. Lancet. (2021) 398:1700–12. doi: 10.1016/S0140-6736(21)02143-7, PMID: 34634250 PMC8500697

[14] Bulhões C Ramos E Dias S Barros H. Depressive symptoms at 13 years as predictors of depression in older adolescents: a prospective 4-year follow-up study in a nonclinical population. Eur Child Adolesc Psychiatry. (2019) 28:595–9. doi: 10.1007/s00787-018-1194-0, PMID: 29974214

[15] Sampasa-Kanyinga H Colman I Goldfield GS Janssen I Wang J Podinic I. Combinations of physical activity, sedentary time, and sleep duration and their associations with depressive symptoms and other mental health problems in children and adolescents: a systematic review. Int J Behav Nutr Phys Act. (2020) 17:72. doi: 10.1186/s12966-020-00976-x, PMID: 32503638 PMC7273653

[16] Makizako H Tsutsumimoto K Doi T Hotta R Nakakubo S Liu-Ambrose T. Effects of exercise and horticultural intervention on the brain and mental health in older adults with depressive symptoms and memory problems: study protocol for a randomized controlled trial [UMIN000018547]. Trials. (2015) 16:499. doi: 10.1186/s13063-015-1032-3, PMID: 26537979 PMC4634591

[17] Osborne LM Yenokyan G Fei K Kraus T Moran T Monk C. Innate immune activation and depressive and anxious symptoms across the peripartum: An exploratory study. Psychoneuroendocrinology. (2019) 99:80–6. doi: 10.1016/j.psyneuen.2018.08.038, PMID: 30195110 PMC6234836

[18] Guloksuz S Wichers M Kenis G Russel MG Wauters A Verkerk R. Depressive symptoms in Crohn’s disease: relationship with immune activation and tryptophan availability. PLoS One. (2013) 8:e60435. doi: 10.1371/journal.pone.0060435, PMID: 23544139 PMC3609785

[19] Zhang MW Ho RC Cheung MW Fu E Mak A. Prevalence of depressive symptoms in patients with chronic obstructive pulmonary disease: a systematic review, meta-analysis and meta-regression. Gen Hosp Psychiatry. (2011) 33:217–23. doi: 10.1016/j.genhosppsych.2011.03.009, PMID: 21601717

[20] O’Toole J Woo H Putcha N Cooper CB Woodruff P Kanner RE. Comparative impact of depressive symptoms and FEV(1)% on chronic obstructive pulmonary disease. Ann Am Thorac Soc. (2022) 19:171–8. doi: 10.1513/AnnalsATS.202009-1187OC, PMID: 34410883 PMC8867359

[21] Coentre R Talina MC Góis C Figueira ML. Depressive symptoms and suicidal behavior after first-episode psychosis: a comprehensive systematic review. Psychiatry Res. (2017) 253:240–8. doi: 10.1016/j.psychres.2017.04.010, PMID: 28395229

[22] Augenstein TM Visser KF Gallagher K De Los RA D’Angelo EJ Nock MK. Multi-informant reports of depressive symptoms and suicidal ideation among adolescent inpatients. Suicide Life Threat Behav. (2022) 52:99–109. doi: 10.1111/sltb.12803, PMID: 34608660

[23] Fortes C Mastroeni S Alessandra S Lindau J Farchi S Franco F. The combination of depressive symptoms and smoking shorten life expectancy among the aged. Int Psychogeriatr. (2012) 24:624–30. doi: 10.1017/S1041610211002201, PMID: 22152085

[24] Damián J Pastor-Barriuso R Valderrama-Gama E de Pedro-Cuesta J. Association of detected depression and undetected depressive symptoms with long-term mortality in a cohort of institutionalised older people. Epidemiol Psychiatr Sci. (2017) 26:189–98. doi: 10.1017/S2045796015001171, PMID: 26753838 PMC6998690

[25] Sowan W Rutin R Cohen M. Chronic stressors, coping strategies, and depressive symptoms: a comparison across older age groups. Stress Health. (2023) 39:1037–46. doi: 10.1002/smi.3237, PMID: 36848591

[26] Fung HW Liu C Yuan GF Liu J Zhao J Chien WT. Association among negative life events, sense of security, and depressive symptoms in Chinese adolescents after the 2013 Ya’an earthquake. Disaster Med Public Health Prep. (2023) 17:e352. doi: 10.1017/dmp.2022.300 36916203

[27] Wang DF Zhou YN Liu YH Hao YZ Zhang JH Liu TQ. Social support and depressive symptoms: exploring stigma and self-efficacy in a moderated mediation model. BMC Psychiatry. (2022) 22:117. doi: 10.1186/s12888-022-03740-6, PMID: 35168584 PMC8845403

[28] Kleppang AL Haugland SH Bakken A Stea TH. Lifestyle habits and depressive symptoms in Norwegian adolescents: a national cross-sectional study. BMC Public Health. (2021) 21:816. doi: 10.1186/s12889-021-10846-1, PMID: 33910539 PMC8082771

[29] Delitala AP Terracciano A Fiorillo E Orrù V Schlessinger D Cucca F. Depressive symptoms, thyroid hormone and autoimmunity in a population-based cohort from Sardinia. J Affect Disord. (2016) 191:82–7. doi: 10.1016/j.jad.2015.11.019, PMID: 26655116 PMC4715961

[30] Kappelmann N Arloth J Georgakis MK Czamara D Rost N Ligthart S. Dissecting the association between inflammation, metabolic dysregulation, and specific depressive symptoms: a genetic correlation and 2-sample Mendelian randomization study. JAMA Psychiatry. (2021) 78:161–70. doi: 10.1001/jamapsychiatry.2020.3436, PMID: 33079133 PMC7577200

[31] Refisch A Sen ZD Klassert TE Busch A Besteher B Danyeli LV. Microbiome and immuno-metabolic dysregulation in patients with major depressive disorder with atypical clinical presentation. Neuropharmacology. (2023) 235:109568. doi: 10.1016/j.neuropharm.2023.109568, PMID: 37182790

[32] Harshfield EL Pennells L Schwartz JE Willeit P Kaptoge S Bell S. Association between depressive symptoms and incident cardiovascular diseases. JAMA. (2020) 324:2396–405. doi: 10.1001/jama.2020.23068, PMID: 33320224 PMC7739139

[33] Mukherjee N Chaturvedi SK. Depressive symptoms and disorders in type 2 diabetes mellitus. Curr Opin Psychiatry. (2019) 32:416–21. doi: 10.1097/YCO.0000000000000528 31135489

[34] Otten D Ernst M Werner AM Tibubos AN Reiner I Brähler E. Depressive symptoms predict the incidence of common chronic diseases in women and men in a representative community sample. Psychol Med. (2023) 53:4172–80. doi: 10.1017/S0033291722000861, PMID: 35443907 PMC10317822

[35] Jiang C Hu C Li D. Linking chronic diseases, cognitive impairment and depressive symptoms in Chinese older adults: a three-wave population-based longitudinal study. J Affect Disord. (2023) 324:496–501. doi: 10.1016/j.jad.2022.12.150, PMID: 36621673

[36] Zhang C Chang Y Yun Q Lu J Zheng X Xue Y. The impact of chronic diseases on depressive symptoms among the older adults: the role of sleep quality and empty nest status. J Affect Disord. (2022) 302:94–100. doi: 10.1016/j.jad.2022.01.090, PMID: 35085671

[37] Huang D Wang J Fang H Fu Y Lou J. Longitudinal association of chronic diseases with depressive symptoms in middle-aged and older adults in China: mediation by functional limitations, social interaction, and life satisfaction. J Glob Health. (2023) 13:04119. doi: 10.7189/jogh.13.04119, PMID: 37766663 PMC10534192

[38] Parajuli J Berish D Jao YL. Chronic conditions and depressive symptoms in older adults: the mediating role of functional limitations. Aging Ment Health. (2021) 25:243–9. doi: 10.1080/13607863.2019.1693971, PMID: 31762296

[39] Leung J Gouda H Chung J Irmansyah I. Comorbidity between depressive symptoms and chronic conditions - findings from the Indonesia family life survey. J Affect Disord. (2021) 280:236–40. doi: 10.1016/j.jad.2020.11.007, PMID: 33220559

[40] Hu J Zheng X Shi G Guo L. Associations of multiple chronic disease and depressive symptoms with incident stroke among Chinese middle-aged and elderly adults: a nationwide population-based cohort study. BMC Geriatr. (2022) 22:660. doi: 10.1186/s12877-022-03329-4, PMID: 35953770 PMC9373457

[41] Chodosh J Miller-Martinez D Aneshensel CS Wight RG Karlamangla AS. Depressive symptoms, chronic diseases, and physical disabilities as predictors of cognitive functioning trajectories in older Americans. J Am Geriatr Soc. (2010) 58:2350–7. doi: 10.1111/j.1532-5415.2010.03171.x, PMID: 21087219 PMC3058867

[42] Nisar A Yin J Waqas A Bai X Wang D Rahman A. Prevalence of perinatal depression and its determinants in mainland China: a systematic review and meta-analysis. J Affect Disord. (2020) 277:1022–37. doi: 10.1016/j.jad.2020.07.046, PMID: 33065811

[43] Tan J Ma C Zhu C Wang Y Zou X Li H. Prediction models for depression risk among older adults: systematic review and critical appraisal. Ageing Res Rev. (2023) 83:101803. doi: 10.1016/j.arr.2022.101803, PMID: 36410622

[44] Yunming L Changsheng C Haibo T Wenjun C Shanhong F Yan M. Prevalence and risk factors for depression in older people in Xi’an China: a community-based study. Int J Geriatr Psychiatry. (2012) 27:31–9. doi: 10.1002/gps.2685, PMID: 21284042

[45] Lu L Shen H Tan L Huang Q Chen Q Liang M. Prevalence and factors associated with anxiety and depression among community-dwelling older adults in Hunan, China: a cross-sectional study. BMC Psychiatry. (2023) 23:107. doi: 10.1186/s12888-023-04583-5, PMID: 36793025 PMC9930706

[46] Yang C Yao T Huang Y Zhao L Zhang L. Prevalence and influencing factors of depression of caregivers in children with epilepsy in southwestern China: a cross-sectional study. Medicine (Baltimore). (2021) 100:e23571. doi: 10.1097/MD.0000000000023571, PMID: 33725809 PMC7969277

[47] Li F Cui Y Li Y Guo L Ke X Liu J. Prevalence of mental disorders in school children and adolescents in China: diagnostic data from detailed clinical assessments of 17,524 individuals. J Child Psychol Psychiatry. (2022) 63:34–46. doi: 10.1111/jcpp.13445, PMID: 34019305

[48] Liu Q Cai H Yang LH Xiang YB Yang G Li H. Depressive symptoms and their association with social determinants and chronic diseases in middle-aged and elderly Chinese people. Sci Rep. (2018) 8:3841. doi: 10.1038/s41598-018-22175-2, PMID: 29497126 PMC5832867

[49] Edwards N Walker S Paddick SM Prina AM Chinnasamy M Reddy N. Prevalence of depression and anxiety in older people in low- and middle- income countries in Africa, Asia and South America: a systematic review and meta-analysis. J Affect Disord. (2023) 325:656–74. doi: 10.1016/j.jad.2023.01.068, PMID: 36681304

[50] Hao Y Farah MJ. Heterogeneity of depression across the socioeconomic spectrum. Proc Natl Acad Sci USA. (2023) 120:e2222069120. doi: 10.1073/pnas.2222069120, PMID: 37036974 PMC10119997

[51] Hong C Xiong X Li J Ning X Qi D Yang Y. Urbanization and depressive symptoms among middle-aged and older adults in China. Front Public Health. (2022) 10:1086248. doi: 10.3389/fpubh.2022.1086248, PMID: 36620302 PMC9816896

[52] Gomes S von Schantz M Leocadio-Miguel M. Predicting depressive symptoms in middle-aged and elderly adults using sleep data and clinical health markers: a machine learning approach. Sleep Med. (2023) 102:123–31. doi: 10.1016/j.sleep.2023.01.002, PMID: 36641929

[53] Wang LY Lin LP Chen YC Wang TW Lin JD. Correlates of depressive symptoms among middle-aged and older homeless adults using the 9-item patient health questionnaire. Int J Environ Res Public Health. (2020) 17:4754. doi: 10.3390/ijerph17134754, PMID: 32630635 PMC7370065

[54] Marques A Henriques-Neto D Peralta M Marconcin P Gouveia ÉR Ferrari G. Exploring grip strength as a predictor of depression in middle-aged and older adults. Sci Rep. (2021) 11:15946. doi: 10.1038/s41598-021-95566-7, PMID: 34354204 PMC8342600

[55] Sjöberg L Karlsson B Atti AR Skoog I Fratiglioni L Wang HX. Prevalence of depression: comparisons of different depression definitions in population-based samples of older adults. J Affect Disord. (2017) 221:123–31. doi: 10.1016/j.jad.2017.06.011, PMID: 28645024

[56] An L Ma L Xu N Yu B. Life satisfaction, depressive symptoms, and blood pressure in the middle-aged and older Chinese population. J Psychosom Res. (2023) 170:111367. doi: 10.1016/j.jpsychores.2023.111367, PMID: 37196586

[57] Miao YF Dong XX Li DL Zhang T Wu Y Pan CW. Chronic conditions and depressive symptoms in middle-aged and older Chinese adults: roles of perceived social support and area of residence. J Affect Disord. (2023) 340:290–8. doi: 10.1016/j.jad.2023.08.045, PMID: 37567346

[58] Liu H Zhou Z Fan X Shen C Ma Y Sun H. Association between multiple chronic conditions and depressive symptoms among older adults in China: evidence from the China health and retirement longitudinal study (CHARLS). Int J Public Health. (2023) 68:1605572. doi: 10.3389/ijph.2023.1605572, PMID: 36938299 PMC10020227

[59] Seo J Choi B Kim S Lee H Oh D. The relationship between multiple chronic diseases and depressive symptoms among middle-aged and elderly populations: results of a 2009 korean community health survey of 156,747 participants. BMC Public Health. (2017) 17:844. doi: 10.1186/s12889-017-4798-2, PMID: 29070021 PMC5657127

[60] Smith L Shin JI Pizzol D López Sánchez GF Soysal P Veronese N. The association of pain with suicidal ideation and suicide attempts with depressive symptoms among adults aged ≥50 years from low- and middle-income countries. Int J Geriatr Psychiatry. (2023) 38:e5962. doi: 10.1002/gps.5962, PMID: 37427854

[61] Tang ST Chen JS Chou WC Lin KC Chang WC Hsieh CH. Prevalence of severe depressive symptoms increases as death approaches and is associated with disease burden, tangible social support, and high self-perceived burden to others. Support Care Cancer. (2016) 24:83–91. doi: 10.1007/s00520-015-2747-0, PMID: 25933701

[62] Fang EF Xie C Schenkel JA Wu C Long Q Cui H. A research agenda for ageing in China in the 21st century (2nd edition): focusing on basic and translational research, long-term care, policy and social networks. Ageing Res Rev. (2020) 64:101174. doi: 10.1016/j.arr.2020.101174, PMID: 32971255 PMC7505078

[63] Fang EF Scheibye-Knudsen M Jahn HJ Li J Ling L Guo H. A research agenda for aging in China in the 21st century. Ageing Res Rev. (2015) 24:197–205. doi: 10.1016/j.arr.2015.08.003, PMID: 26304837 PMC5179143

[64] Molyneaux E Pasupathy D Kenny LC McCowan L North RA Dekker GA. Socio-economic status influences the relationship between obesity and antenatal depression: data from a prospective cohort study. J Affect Disord. (2016) 202:124–7. doi: 10.1016/j.jad.2016.05.061, PMID: 27262633 PMC4957541

[65] Meltzer H Bebbington P Brugha T Jenkins R McManus S Stansfeld S. Job insecurity, socio-economic circumstances and depression. Psychol Med. (2010) 40:1401–7. doi: 10.1017/S0033291709991802, PMID: 19903366

[66] Wang R Wang J Hu S. Study on the relationship of depression, anxiety, lifestyle and eating habits with the severity of reflux esophagitis. BMC Gastroenterol. (2021) 21:127. doi: 10.1186/s12876-021-01717-5, PMID: 33743601 PMC7980552

[67] Kunugi H. Depression and lifestyle: focusing on nutrition, exercise, and their possible relevance to molecular mechanisms. Psychiatry Clin Neurosci. (2023) 77:420–33. doi: 10.1111/pcn.13551, PMID: 36992617 PMC11488618

[68] Moitra P Madan J Shaikh NI. Eating habits and sleep patterns of adolescents with depression symptoms in Mumbai, India. Matern Child Nutr. (2020) 16:e12998. doi: 10.1111/mcn.12998, PMID: 33347724 PMC7752132

[69] Park C Rosenblat JD Brietzke E Pan Z Lee Y Cao B. Stress, epigenetics and depression: a systematic review. Neurosci Biobehav Rev. (2019) 102:139–52. doi: 10.1016/j.neubiorev.2019.04.010, PMID: 31005627

[70] Kiecolt-Glaser JK Derry HM Fagundes CP. Inflammation: depression fans the flames and feasts on the heat. Am J Psychiatry. (2015) 172:1075–91. doi: 10.1176/appi.ajp.2015.15020152, PMID: 26357876 PMC6511978

[71] Opie RS Itsiopoulos C Parletta N Sanchez-Villegas A Akbaraly TN Ruusunen A. Dietary recommendations for the prevention of depression. Nutr Neurosci. (2017) 20:161–71. doi: 10.1179/1476830515Y.0000000043 26317148

[72] Womack LS Rossen LM Hirai AH. Urban-rural infant mortality disparities by race and ethnicity and cause of death. Am J Prev Med. (2020) 58:254–60. doi: 10.1016/j.amepre.2019.09.010, PMID: 31735480 PMC6980981

[73] Wu Y Zheng H Liu Z Wang S Chen X Yu H. Depression and anxiety-free life expectancy by sex and urban-rural areas in Jiangxi, China in 2013 and 2018. Int J Environ Res Public Health. (2021) 18:1991. doi: 10.3390/ijerph18041991, PMID: 33670818 PMC7922042

[74] McFarland DC Doherty M Atkinson TM O’Hanlon R Breitbart W Nelson CJ. Cancer-related inflammation and depressive symptoms: systematic review and meta-analysis. Cancer. (2022) 128:2504–19. doi: 10.1002/cncr.34193, PMID: 35417925 PMC9177733

[75] Burns RJ Briner E Schmitz N. Trajectories of depressive symptoms and incident diabetes: a prospective study. Ann Behav Med. (2022) 56:311–6. doi: 10.1093/abm/kaab094, PMID: 34791017

[76] Markun S Gravestock I Jäger L Rosemann T Pichierri G Burgstaller JM. Effects of vitamin B12 supplementation on cognitive function, depressive symptoms, and fatigue: a systematic review, Meta-analysis, and Meta-regression. Nutrients. (2021) 13:923. doi: 10.3390/nu13030923, PMID: 33809274 PMC8000524

[77] Polenick CA Birditt KS Turkelson A Bugajski BC Kales HC. Discordant chronic conditions and depressive symptoms: longitudinal associations among middle-aged and older couples. J Gerontol B Psychol Sci Soc Sci. (2021) 76:451–60. doi: 10.1093/geronb/gbz137, PMID: 31792532 PMC7887728

[78] Lu W Pai M Scholes S Xue B. Do depressive symptoms link chronic diseases to cognition among older adults? Evidence from the health and retirement study in the United States. J Affect Disord. (2021) 294:357–65. doi: 10.1016/j.jad.2021.07.012, PMID: 34315097

[79] Quiñones AR Hwang J Heintzman J Huguet N Lucas JA Schmidt TD. Trajectories of chronic disease and multimorbidity among middle-aged and older patients at community health centers. JAMA Netw Open. (2023) 6:e237497. doi: 10.1001/jamanetworkopen.2023.7497 37040114 PMC10091154

[80] Solé-Auró A Michaud PC Hurd M Crimmins E. Disease incidence and mortality among older Americans and Europeans. Demography. (2015) 52:593–611. doi: 10.1007/s13524-015-0372-7, PMID: 25715676 PMC4441205

[81] Staunstrup LM Bager CL Frederiksen P Helge JW Brunak S Christiansen C. Endotrophin is associated with chronic multimorbidity and all-cause mortality in a cohort of elderly women. EBioMedicine. (2021) 68:103391. doi: 10.1016/j.ebiom.2021.103391, PMID: 34044221 PMC8167215

[82] Zhao Y Hu Y Smith JP Strauss J Yang G. Cohort profile: the China health and retirement longitudinal study (CHARLS). Int J Epidemiol. (2014) 43:61–8. doi: 10.1093/ije/dys203, PMID: 23243115 PMC3937970

[83] Pu L Zhang J He X Pan D Wang H Zhang X. Association of living arrangements with all-cause mortality among older adults: a propensity score-matched cohort study. BMC Public Health. (2023) 23:1821. doi: 10.1186/s12889-023-16749-7, PMID: 37726743 PMC10508011

[84] Fan Y He D. Self-rated health, socioeconomic status and all-cause mortality in Chinese middle-aged and elderly adults. Sci Rep. (2022) 12:9309. doi: 10.1038/s41598-022-13502-9, PMID: 35662273 PMC9166789

[85] Malakouti SK Pachana NA Naji B Kahani S Saeedkhani M. Reliability, validity and factor structure of the CES-D in Iranian elderly. Asian J Psychiatr. (2015) 18:86–90. doi: 10.1016/j.ajp.2015.08.007, PMID: 26442988

[86] González P Nuñez A Merz E Brintz C Weitzman O Navas EL. Measurement properties of the Center for Epidemiologic Studies Depression Scale (CES-D 10): findings from HCHS/SOL. Psychol Assess. (2017) 29:372–81. doi: 10.1037/pas0000330, PMID: 27295022 PMC5154787

[87] Baron EC Davies T Lund C. Validation of the 10-item Centre for Epidemiological Studies Depression Scale (CES-D-10) in Zulu, Xhosa and Afrikaans populations in South Africa. BMC Psychiatry. (2017) 17:6. doi: 10.1186/s12888-016-1178-x, PMID: 28068955 PMC5223549

[88] Shifren K Anzaldi K. Optimism, well-being, depressive symptoms, and perceived physical health: a study among stroke survivors. Psychol Health Med. (2018) 23:46–57. doi: 10.1080/13548506.2017.1325505, PMID: 28475351

[89] He Y Yang L Zhu X Wu B Zhang S Qian C. Mental health Chatbot for young adults with depressive symptoms during the COVID-19 pandemic: single-blind, three-arm randomized controlled trial. J Med Internet Res. (2022) 24:e40719. doi: 10.2196/40719, PMID: 36355633 PMC9680932

[90] Lyu Y Forsyth A Worthington S. Built environment and self-rated health: comparing young, middle-aged, and older people in Chengdu, China. HERD. (2021) 14:229–46. doi: 10.1177/1937586720982566, PMID: 33397148 PMC8212390

[91] Hinchliff S Gott M. Seeking medical help for sexual concerns in mid- and later life: a review of the literature. J Sex Res. (2011) 48:106–17. doi: 10.1080/00224499.2010.548610, PMID: 21409708

[92] Edwards J Markert R Bricker D. Discharge against medical advice: how often do we intervene. J Hosp Med. (2013) 8:574–7. doi: 10.1002/jhm.2087, PMID: 24101542

[93] Pham B Rios P Radhakrishnan A Darvesh N Antony J Williams C. Comparative-effectiveness research of COVID-19 treatment: a rapid scoping review. BMJ Open. (2022) 12:e045115. doi: 10.1136/bmjopen-2020-045115, PMID: 35947494 PMC9170799

[94] Shimada H Park H Makizako H Doi T Lee S Suzuki T. Depressive symptoms and cognitive performance in older adults. J Psychiatr Res. (2014) 57:149–56. doi: 10.1016/j.jpsychires.2014.06.004 25023083

[95] Ma W Wu B Gao X Zhong R. Association between frailty and cognitive function in older Chinese people: a moderated mediation of social relationships and depressive symptoms. J Affect Disord. (2022) 316:223–32. doi: 10.1016/j.jad.2022.08.032, PMID: 35988782

[96] Jeon HJ Walker RS Inamori A Hong JP Cho MJ Baer L. Differences in depressive symptoms between Korean and American outpatients with major depressive disorder. Int Clin Psychopharmacol. (2014) 29:150–6. doi: 10.1097/YIC.0000000000000019, PMID: 24323201

[97] Touron E Moulinet I Kuhn E Sherif S Ourry V Landeau B. Depressive symptoms in cognitively unimpaired older adults are associated with lower structural and functional integrity in a frontolimbic network. Mol Psychiatry. (2022) 27:5086–95. doi: 10.1038/s41380-022-01772-8, PMID: 36258017 PMC9763117

[98] Lim S Ali SH Mohaimin S Dhar R Dhar M Rahman F. Help seeking and mental health outcomes among south Asian young adult survivors of sexual violence in the New York state region. BMC Public Health. (2022) 22:1147. doi: 10.1186/s12889-022-13489-y 35676672 PMC9174918

[99] Paljärvi T Tiihonen J Lähteenvuo M Tanskanen A Fazel S Taipale H. Mortality in psychotic depression: 18-year follow-up study. Br J Psychiatry. (2023) 222:37–43. doi: 10.1192/bjp.2022.140, PMID: 36250518 PMC10895511

[100] Korous KM Bradley RH Prattley J Luthar SS Li L Levy R. Socioeconomic status and depressive symptoms: An individual-participant data meta-analysis on range restriction and measurement in the United States. J Affect Disord. (2022) 4:50–8. doi: 10.1016/j.jad.2022.06.090 PMID: 35798179 PMC10947555

[101] Purtle J Nelson KL Yang Y Langellier B Stankov I Diez Roux AV. Urban-rural differences in older adult depression: a systematic review and Meta-analysis of comparative studies. Am J Prev Med. (2019) 56:603–13. doi: 10.1016/j.amepre.2018.11.008, PMID: 30777704

[102] Ma T Jin H Kwok LY Sun Z Liong MT Zhang H. Probiotic consumption relieved human stress and anxiety symptoms possibly via modulating the neuroactive potential of the gut microbiota. Neurobiol Stress. (2021) 14:100294. doi: 10.1016/j.ynstr.2021.100294, PMID: 33511258 PMC7816019

[103] Colon-Rivera H Dixon LB. Mental health Services in Rural Areas. Psychiatr Serv. (2020) 71:984–5. doi: 10.1176/appi.ps.71903 32867613

[104] Mufson L Rynn MA. Primary care: meeting the mental health care needs of adolescents with depression. J Am Acad Child Adolesc Psychiatry. (2019) 58:389–91. doi: 10.1016/j.jaac.2018.10.014, PMID: 30926071

[105] Westerlund A Ivarsson A Richter-Sundberg L. Evidence-based practice in child and adolescent mental health services - the challenge of implementing national guidelines for treatment of depression and anxiety. Scand J Caring Sci. (2021) 35:476–84. doi: 10.1111/scs.12859, PMID: 32323362

[106] Madrid Miles CI Bates BR Casapulla SL Grijalva MJ. Social support in rural communities in Manabi province, Ecuador. Rural Remote Health. (2022) 22:6957. doi: 10.22605/RRH6957, PMID: 36328965

[107] Rowland B Swami N Prattley J Duffy J Macdonald JA Perales F. Depressive symptoms and social support among Australian men: a 7-year longitudinal study. Aust N Z J Psychiatry. (2023) 57:1243–52. doi: 10.1177/00048674221151000, PMID: 36717775

[108] Nooijen C Blom V Ekblom Ö Ekblom MM Kallings LV. Improving office workers’ mental health and cognition: a 3-arm cluster randomized controlled trial targeting physical activity and sedentary behavior in multi-component interventions. BMC Public Health. (2019) 19:266. doi: 10.1186/s12889-019-6589-4 30836957 PMC6402109

[109] Magee W Clarke P. The effect of smoking on depressive symptoms. Addict Behav. (2021) 112:106641. doi: 10.1016/j.addbeh.2020.106641 33010527

[110] Kaila-Kangas L Koskinen A Pensola T Mäkelä P Leino-Arjas P. Alcohol-induced morbidity and mortality by occupation: a population-based follow-up study of working Finns. Eur J Pub Health. (2016) 26:116–22. doi: 10.1093/eurpub/ckv145, PMID: 26250707

[111] Zhang Z Jackson SL Gillespie C Merritt R Yang Q. Depressive symptoms and mortality among US adults. JAMA Netw Open. (2023) 6:e2337011. doi: 10.1001/jamanetworkopen.2023.37011, PMID: 37812418 PMC10562940

[112] Shaygan M Yazdanpanah M. Depression and work-family conflict mediating the effects of job stress on chronic pain: a structural equation modelling approach. Int J Occup Saf Ergon. (2022) 28:2551–8. doi: 10.1080/10803548.2021.2008130, PMID: 34789081

[113] Li H Zheng D Li Z Wu Z Feng W Cao X. Association of Depressive Symptoms with Incident Cardiovascular Diseases in middle-aged and older Chinese adults. JAMA Netw Open. (2019) 2:e1916591. doi: 10.1001/jamanetworkopen.2019.16591, PMID: 31800066 PMC6902756

[114] Chiang JJ Cole SW Bower JE Irwin MR Taylor SE Arevalo J. Depressive symptoms and immune transcriptional profiles in late adolescents. Brain Behav Immun. (2019) 80:163–9. doi: 10.1016/j.bbi.2019.03.004, PMID: 30851376 PMC6710012

[115] Andrade D Rocha RM Ribeiro ÍJS. Depressive symptoms among older adults with diabetes mellitus: a cross-sectional study. Sao Paulo Med J. (2022) 141:e2021771. doi: 10.1590/1516-3180.2021.0771.R5.09082022 36197348 PMC10065091

[116] Sun M Wang L Hu Y Wang X Yan S Guo Y. Cognitive impairment mediates the association between dietary inflammation and depressive symptoms in the elderly. Nutrients. (2022) 14:5118. doi: 10.3390/nu14235118, PMID: 36501149 PMC9737219

[117] Vance DE. Speed of processing in older adults: a cognitive overview for nursing. J Neurosci Nurs. (2009) 41:290–7. doi: 10.1097/jnn.0b013e3181b6beda 19998680

[118] Zhong BL Ruan YF Xu YM Chen WC Liu LF. Prevalence and recognition of depressive disorders among Chinese older adults receiving primary care: a multi-center cross-sectional study. J Affect Disord. (2020) 260:26–31. doi: 10.1016/j.jad.2019.09.011, PMID: 31493635

[119] Fang J Zhang Z Greenlund KJ. Association of depressive symptoms and hypertension prevalence, awareness, treatment and control among USA adults. J Hypertens. (2022) 40:1658–65. doi: 10.1097/HJH.0000000000003163, PMID: 35822590 PMC11139467

[120] Dauphinot V Rouch I Kossovsky MP Pichot V Dorey JM Krolak-Salmon P. Depressive symptoms and autonomic nervous system dysfunction in an elderly population-based study: the PROOF study. J Affect Disord. (2012) 143:153–9. doi: 10.1016/j.jad.2012.05.045, PMID: 22910448

[121] Saugel B Kouz K Scheeren T Greiwe G Hoppe P Romagnoli S. Cardiac output estimation using pulse wave analysis-physiology, algorithms, and technologies: a narrative review. Br J Anaesth. (2021) 126:67–76. doi: 10.1016/j.bja.2020.09.049, PMID: 33246581

[122] Hantsoo L Jagodnik KM Novick AM Baweja R di Scalea TL Ozerdem A. The role of the hypothalamic-pituitary-adrenal axis in depression across the female reproductive lifecycle: current knowledge and future directions. Front Endocrinol (Lausanne). (2023) 14:1295261. doi: 10.3389/fendo.2023.1295261, PMID: 38149098 PMC10750128

[123] Incollingo Rodriguez AC Epel ES White ML Standen EC Seckl JR Tomiyama AJ. Hypothalamic-pituitary-adrenal axis dysregulation and cortisol activity in obesity: a systematic review. Psychoneuroendocrinology. (2015) 62:301–18. doi: 10.1016/j.psyneuen.2015.08.014, PMID: 26356039

[124] Haddad C Courand PY Berge C Harbaoui B Lantelme P. Impact of cortisol on blood pressure and hypertension-mediated organ damage in hypertensive patients. J Hypertens. (2021) 39:1412–20. doi: 10.1097/HJH.0000000000002801 33534343

[125] Villarreal-Zegarra D Bernabe-Ortiz A. Association between arterial hypertension and depressive symptoms: results from population-based surveys in Peru. Asia Pac Psychiatry. (2020) 12:e12385. doi: 10.1111/appy.12385, PMID: 32119760

[126] Im EO Yi JS Chee W. Depressive symptoms and type II diabetes mellitus among midlife women. Menopause. (2021) 28:650–9. doi: 10.1097/GME.0000000000001759 33739318 PMC7951177

[127] Cole JB Florez JC. Genetics of diabetes mellitus and diabetes complications. Nat Rev Nephrol. (2020) 16:377–90. doi: 10.1038/s41581-020-0278-5, PMID: 32398868 PMC9639302

[128] Ceriello A Prattichizzo F. Variability of risk factors and diabetes complications. Cardiovasc Diabetol. (2021) 20:101. doi: 10.1186/s12933-021-01289-4, PMID: 33962641 PMC8106175

[129] Lau LH Lew J Borschmann K Thijs V Ekinci EI. Prevalence of diabetes and its effects on stroke outcomes: a meta-analysis and literature review. J Diabetes Investig. (2019) 10:780–92. doi: 10.1111/jdi.12932, PMID: 30220102 PMC6497593

[130] Jankauskas SS Kansakar U Varzideh F Wilson S Mone P Lombardi A. Heart failure in diabetes. Metabolism. (2021) 125:154910. doi: 10.1016/j.metabol.2021.154910, PMID: 34627874 PMC8941799

[131] Mancusi C Izzo R di Gioia G Losi MA Barbato E Morisco C. Insulin resistance the hinge between hypertension and type 2 diabetes. High Blood Press Cardiovasc Prev. (2020) 27:515–26. doi: 10.1007/s40292-020-00408-8, PMID: 32964344 PMC7661395

[132] Qiao T Luo T Pei H Yimingniyazi B Aili D Aimudula A. Association between abdominal obesity indices and risk of cardiovascular events in Chinese populations with type 2 diabetes: a prospective cohort study. Cardiovasc Diabetol. (2022) 21:225. doi: 10.1186/s12933-022-01670-x, PMID: 36320060 PMC9628026

[133] Yuge H Okada H Hamaguchi M Kurogi K Murata H Ito M. Triglycerides/HDL cholesterol ratio and type 2 diabetes incidence: Panasonic cohort study 10. Cardiovasc Diabetol. (2023) 22:308. doi: 10.1186/s12933-023-02046-5, PMID: 37940952 PMC10634002

[134] Davis MC Lemery-Chalfant K Yeung EW Luecken LJ Zautra AJ Irwin MR. Interleukin-6 and depressive mood symptoms: mediators of the association between childhood abuse and cognitive performance in middle-aged adults. Ann Behav Med. (2019) 53:29–38. doi: 10.1093/abm/kay014, PMID: 29562248 PMC6301312

[135] Lotrich F. Depression symptoms, low-grade inflammatory activity, and new targets for clinical intervention. Biol Psychiatry. (2011) 70:111–2. doi: 10.1016/j.biopsych.2011.05.022, PMID: 21708303

[136] Postal M Lapa AT Sinicato NA de Oliveira PK Peres FA Costallat LT. Depressive symptoms are associated with tumor necrosis factor alpha in systemic lupus erythematosus. J Neuroinflammation. (2016) 13:5. doi: 10.1186/s12974-015-0471-9, PMID: 26732584 PMC4702302

[137] Conforti A Di Cola I Pavlych V Ruscitti P Berardicurti O Ursini F. Beyond the joints, the extra-articular manifestations in rheumatoid arthritis. Autoimmun Rev. (2021) 20:102735. doi: 10.1016/j.autrev.2020.102735, PMID: 33346115

[138] Khir N Noh A Long I Ismail NI Siran R Ismail C. Inflammatory-associated apoptotic markers: are they the culprit to rheumatoid arthritis pain. Mol Biol Rep. (2022) 49:10077–90. doi: 10.1007/s11033-022-07591-y, PMID: 35699858

[139] Conigliaro P Chimenti MS Triggianese P Sunzini F Novelli L Perricone C. Autoantibodies in inflammatory arthritis. Autoimmun Rev. (2016) 15:673–83. doi: 10.1016/j.autrev.2016.03.003 26970491

[140] Duggleby W Raudonis BM. Dispelling myths about palliative care and older adults. Semin Oncol Nurs. (2006) 22:58–64. doi: 10.1016/j.soncn.2005.10.008, PMID: 16458184

[141] Visser RC MacInnes D Parrott J Houben F. Growing older in secure mental health care: the user experience. J Ment Health. (2021) 30:51–7. doi: 10.1080/09638237.2019.1630722, PMID: 31257967

[142] Kong D Solomon P Dong X. Comorbid depressive symptoms and chronic medical conditions among US Chinese older adults. J Am Geriatr Soc. (2019) 67:S545–50. doi: 10.1111/jgs.15669, PMID: 31403205

[143] Neuhouser ML. The importance of healthy dietary patterns in chronic disease prevention. Nutr Res. (2019) 70:3–6. doi: 10.1016/j.nutres.2018.06.002, PMID: 30077352 PMC6328339

[144] Jang Y Park NS Park J Chiriboga DA Haley WE Kim MT. The mental health benefit of friend networks in older Korean Americans: the conditioning effect of family type. J Gerontol B Psychol Sci Soc Sci. (2023) 78:143–53. doi: 10.1093/geronb/gbac109, PMID: 35961306 PMC9890900

[145] Laliberté Durish C Pereverseff RS Yeates KO. Depression and depressive symptoms in pediatric traumatic brain injury: a scoping review. J Head Trauma Rehabil. (2018) 33:E18–30. doi: 10.1097/HTR.0000000000000343, PMID: 28926485 PMC5857396

[146] James C Powell M Seixas A Bateman A Pengpid S Peltzer K. Exploring the psychometric properties of the CES-D-10 and its practicality in detecting depressive symptomatology in 27 low- and middle-income countries. Int J Psychol. (2020) 55:435–45. doi: 10.1002/ijop.12613, PMID: 31441518

