# Supplementary figures and images for: Depressive symptoms and all-cause mortality among middle-aged and older people in China and associations with chronic diseases
Source: Front Public Health. 2024 May 22;12:1381273. doi: 10.3389/fpubh.2024.1381273 (PMC11151855; doi:10.3389/fpubh.2024.1381273)

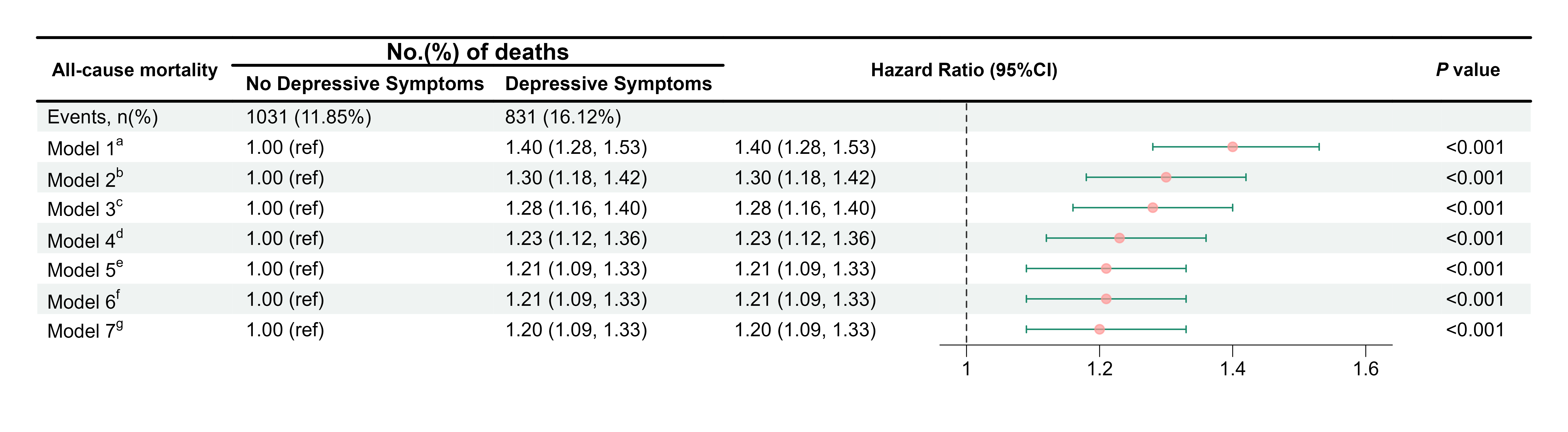

Supplement: Supplementary file 1 [file Presentation_1.zip › eFigure 2.tiff]
